# Different stability of miRNAs and endogenous control genes in archival specimens of papillary thyroid carcinoma

**DOI:** 10.1186/s10020-020-00218-7

**Published:** 2020-11-05

**Authors:** Daina Pamedytyte, Enrika Leipute, Birute Zilaitiene, Valdas Sarauskas, Dalia Dauksiene, Albertas Dauksa, Aurelija Zvirbliene

**Affiliations:** 1 Institute of Biotechnology, Life Sciences Center, Vilnius University, Sauletekio Av. 7, 10257 Vilnius, Lithuania; 2 Department of Pathology, Lithuanian University of Health Sciences, Eiveniu Str. 2, 50161 Kaunas, Lithuania; 3 Institute of Endocrinology, Medical Academy, Lithuanian University of Health Sciences, Eiveniu Str. 2, 50161 Kaunas, Lithuania; 4 Institute for Digestive Research, Medical Academy, Lithuanian University of Health Sciences, Eiveniu Str. 2, 50161 Kaunas, Lithuania

**Keywords:** Stability, miRNA, Endogenous control, FFPE, Papillary thyroid carcinoma

## Abstract

**Background:**

The most popular miRNA quantitation technique is RQ-PCR with relative gene expression method that requires an endogenous control (EC) gene for data normalization. However, there are insufficient data and selection criteria on the most suitable ECs for miRNA expression studies in many cancer types including papillary thyroid carcinoma (PTC). Therefore, in this study we evaluated the impact of chosen EC and archival formalin-fixed, paraffin-embedded (FFPE) PTC tissue age on estimated target miRNA expression.

**Methods:**

RQ-PCR was used to determine expression levels of five miRNAs (miR-146b, miR-222, miR-21, miR-221 and miR-181b) and three different endogenous controls (RNU48, let-7a, miR-16), which were used to normalize the data. In total, 400 FFPE PTC tissues were analyzed that have been stored from 1 to 15 years.

**Results:**

The stability of commonly used ECs RNU48 and let-7a significantly differs from the stability of target miRNA in archival FFPE PTC tissues. Moreover, these differences have a great impact on miRNA expression results when FFPE tissue samples have been stored for a different period of time.

**Conclusions:**

It is important to select an ECs not only stable in the tissue of interest but also with similar stability to target miRNA, especially when working with samples of different age.

## Background

MicroRNAs (miRNAs) are small non-coding RNAs that play important regulatory roles by targeting mRNAs for cleavage or translational repression (Bartel 2004). Disregulated miRNA expression has been reported in many human cancer cells including thyroid cancer. Papillary thyroid carcinoma (PTC) is the most common type of thyroid cancer and it was shown by many researchers that numerous miRNAs are aberrantly expressed during disease development or progression, making these miRNAs attractive as diagnostic or prognostic biomarkers (Leonardi et al. 2012). The ability to use specific miRNAs as biomarkers would assist in determining disease recurrence risk based on the miRNA expression in primary tumor and therefore would allow stratifying patients for a more personalized treatment.

Currently the most popular method for miRNA quantitation is real-time quantitative PCR (RQ-PCR) due to its sensitivity and reproducibility. In order to get reliable data on relative miRNA expression by RQ-PCR, corrections must be made for variation between reactions introduced during all experimental steps from RNA purification to the miRNA amplification. Normalizing the data with endogenous control (EC) gene is widely used method but candidate ECs must be proven to be stably-expressed across the test sample set. The use of unreliable and not properly tested ECs may lead to inaccurate results (Wong and Medrano 2005; McNeill et al. 2007; Davoren et al. 2008). However, a thorough research on the most suitable ECs for miRNA expression normalizations in specific tissues is lacking. To our best knowledge, there are no published data on the most suitable ECs for miRNA expression normalization in thyroid tissue. Different ECs are used by different authors usually with little or no information on why the particular EC was chosen. This also can be one of the reasons why miRNA expression results in PTC differs substantially between the studies.

Archival formalin-fixed, paraffin-embedded (FFPE) tissues represent a vital and convenient resource for gene expression studies in clinical molecular biology research. These samples provide a preserved information of tissue morphology, cytological details, protein and nucleic acid expression that can be later associated with disease outcomes. Properly prepared FFPE tissue samples can be stored at room temperature for many years and keep the structural integrity and RNA expression profile of the initial tissue. However, FFPE sample preparation and their long-term storage may result in damage and partial degradation of DNA and RNA transcripts (Turashvili et al. 2012; Cronin et al. 2004; Peskoe et al. 2017).

Measuring RNA integrity number (RIN) score is one of the methods to asses RNA quality in order to exclude the most degraded and presumably unreliable specimens from gene expression analysis (Schroeder et al. 2006). However, it remains unclear how well the analyses of general RNA transcript size represent the quality or stability of small non-coding RNA, such as miRNAs which are shown to be unusually stable when compared to longer mRNA transcripts. In fact, recent studies show (Peskoe et al. 2017) that there is no association between sample RIN and FFPE block age while detected miRNA expression levels are highly dependent on FFPE block age. Moreover, no statistically significant correlation was found between RIN and RNU6B snRNA, miR-21, or miR-221 transcript levels (Peskoe et al. 2017). These results reflect the lack of knowledge on the stability of miRNAs or other small RNAs and demonstrate the absence of appropriate tools for assessing miRNA quality in clinical specimens. For this reason, there is a need to investigate the stability of miRNAs and the currently used ECs in aged FFPE samples.

In order to reveal the association between the age of FFPE tissue and the expression pattern of the selected ECs and target miRNAs, we have analyzed 400 well-documented FFPE PTC tissues prepared between 2003 and 2017. This study was performed on samples of a single tissue type that were isolated and processed by a single institution. Therefore, tissue type, processing methodology and storage have been consistent. Using the RQ-PCR with relative gene expression method we compared miRNA expression levels in older and fresher FFPE PTC tissues when different ECs are used to normalize the data. We have further analyzed the stability of individual target miRNAs and ECs to evaluate how well these molecules are preserved during a prolonged storage in FFPE blocks and how their storage time could influence the measured expression level of the target miRNA.

## Methods

### Tissue samples

The formalin-fixed, paraffin-embedded (FFPE) PTC tissues were obtained from patients (aged from 10 to 83) who underwent total thyroidectomy at the Lithuanian University of Health Sciences Kauno klinikos in 2003–2017. The study was approved by the Kaunas Regional Committee of Biomedical Research (Lithuania, Permission No. BE-2-44; 2015-12-23). The written informed consent has been obtained from each participant of the study after full explanation of the purpose and nature of all procedures used. This study was conducted in accordance with the Declaration of Helsinki. In total, 400 PTC samples were included in the study.

### RNA extraction

Total RNA was extracted from 5 to 10 mm^3^ sections of FFPE PTC tissues using the miRNeasy FFPE Kit (Qiagen, Hilden, Germany) according to the manufacturer ‘s protocols and stored at − 70 °C. The PTC tissue samples were reviewed by pathologist. Three sections (10 μm) were cut with a micro-tome from each FFPE block. After staining with hematoxylin and eosin, cancer tissue was microdissected under a microscope (×100 magnification) from each section. The PTC cell cellularity was at least 90% in all samples. RNA concentration and quality were examined by NanoDrop 2000 Spectrophotometer (ThermoFisher Scientific, Waltham, MA USA).

### Reverse transcription (RT) and real-time quantitative PCR (RQ-PCR)

Reverse transcription and RQ-PCR were performed using the TaqMan miRNA Reverse Transcription Kit (Applied Biosystems, Foster City, USA), TaqMan miRNA assays (Applied Biosystems, Foster City, USA) and TaqMan Universal PCR Master Mix (Applied Biosystems, Foster City, USA). Briefly, 20 ng of purified tumor-derived RNA was applied to RT and RQ-PCR was performed using Rotor-Gene 6000 PCR system (Corbett Research, Hilden, Germany) following the manufacturer ‘s protocols. The relative expression level was assessed using ΔΔCt method, where ΔCt is the difference in threshold cycles for target and reference genes. The fold expression changes were calculated by the 2^−ΔCt^ method (Schmittgen and Livak 2008). Three RQ-PCR reactions were run per sample according to the manufacturer’s instructions. The limit of confidence for the Ct was 0.5.

### Statistical analysis

The data were analyzed using RStudio and Microsoft Excel (2013) programs. TheNormFinder (Version 0.953) algorithm was used as an Excel add-in (available at https://moma.dk/normfinder-software) (Andersen et al. 2004). For the comparison of miRNA expression levels between the analyzed groups, t-test was used for normally distributed variables and Mann–Whitney U test was used when the distribution of data was not normal. Data normality was tested using the Shapiro–Wilk test. Correlation test was used to determine the significance of linear correlation between measured Ct value and sample age in years. The p-value of 0.05 was considered statistically significant.

## Results and discussion

### The most popular endogenous controls used in PTC research

As the first step in our research, we have analyzed the currently available published data on the ECs used for miRNA profiling in PTC studies. The analysis of previous reports shows that the most popular controls for miRNA research are small nuclear or nucleolar RNAs, such as U6 (Pallante et al. 2006; Chou et al. 2010; Chen et al. 2008; Dettmer et al. 2013; Sun et al. 2013; Tetzlaff et al. 2007), RNU48 (Sheu et al. 2009, 2010; Lee et al. 2013; Sondermann et al. 2015; Dai et al. 2017) and RNU44 (Dettmer et al. 2013; Nikiforova et al. 2008; Linwah et al. 2011). In several studies, miRNAs such as let-7a (Nikiforova et al. 2008; Agretti et al. 2012) and miR-16 (Han et al. 2016) were used as ECs for miRNA quantification in PTC specimens.

### The levels of miRNA expression are different in tissue samples of different age

Next, we investigated how specific ECs can influence the results of the measured miRNA expression levels and evaluated whether the results of miRNA profiling depend on the FFPE PTC tissue sample age. The specimens of PTC tissues have been collected at the Lithuanian University of Health Sciences (Kaunas, Lithuania) over several decades and underwent standardized processing, fixation, and storage procedures. Therefore, these specimens represent a unique and well-controlled resource for miRNA stability analyses.

**Fig. 1:**
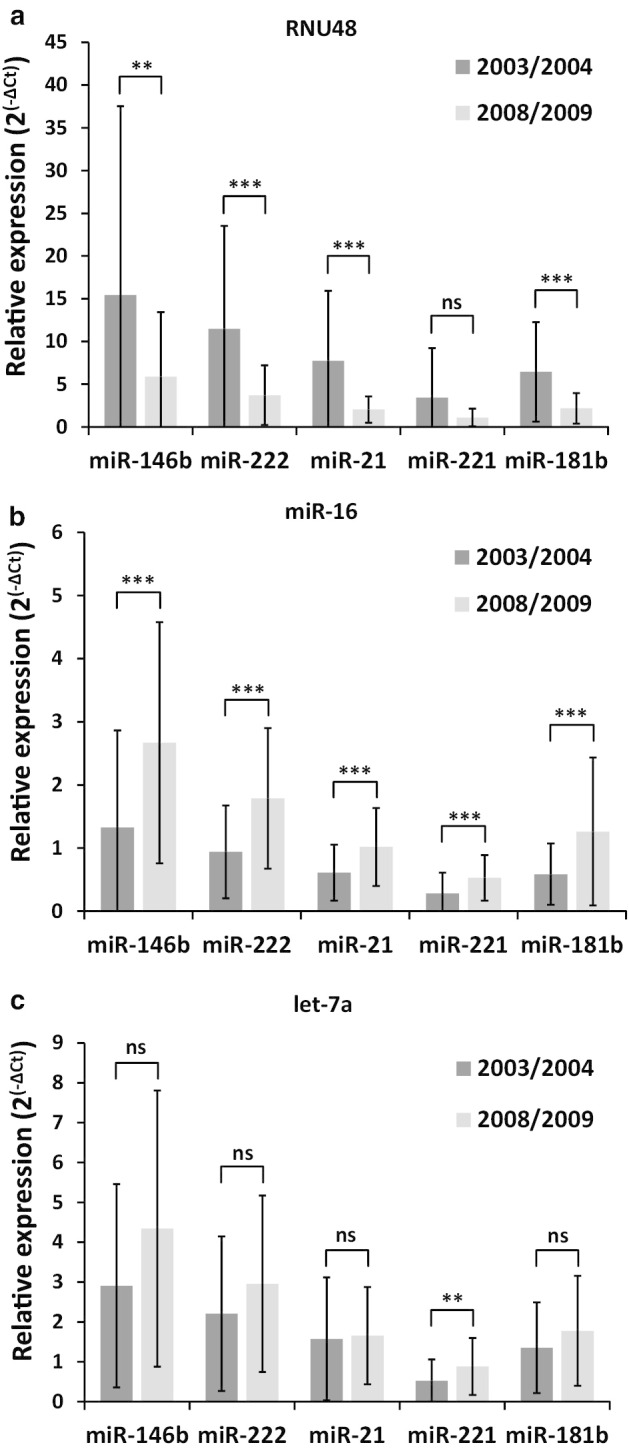
Differences in miR-146b, miR-222, miR-21, miR-221, miR-181b expression levels between FFPE PTC tissue samples prepared in 2003, 2004 (n = 46) and 2008, 2009 (n = 31) when normalized with different ECs: **a** RNU48, **b** miR-16 and **c** let-7a. Relative expression of miR-181b shown in the graphs was multiplied by 2000. All data are presented as the mean ± SD. *p < 0.05, **p < 0.01, and ***p < 0.001. *ns* not significant

In order to evaluate the association between the storage time of FFPE specimens and miRNA profiling results, we have selected 77 FFPE PTC specimens and divided them according to their age. Samples prepared in 2003 and 2004 were placed in one group (n = 46) and samples prepared in 2008 and 2009 were placed in another group (n = 31). The expression pattern of five miRNAs often overexpressed in PTC (miR-146b, -222, -21, -221 and -181b) and 3 ECs (RNU48, let-7a and miR-16) was analyzed. It was clearly demonstrated that the levels of measured miRNA expression differ in fresher (samples prepared in 2008 and 2009) and older (2003 and 2004) PTC tissues when normalized with different ECs. When miR-146b, miR-222, miR-21, miR-221 and miR-181b expression was normalized with RNU48, the expression levels of these miRNAs seemed to be much higher in older PTC samples as compared to fresher samples (p = 0.005; p < 0.001; p < 0.001; p = 0.072; p < 0.001, respectively) (Fig. 1a). Interestingly, when miRNA expression was normalized with either let-7a or miR-16, we noticed a different expression pattern—the levels of miRNA expression were higher in samples prepared in 2008 and 2009 (Fig. 1b, c). The difference in miRNA expression levels between the groups when normalized with miR-16 was statistically significant (p < 0.001 for all miRNAs). In contrast, when let-7a was selected as an EC, it normalized the miRNA expression much better. Although miRNA expression levels normalized with let-7a were slightly higher in fresher samples, the differences between the groups were mostly not significant (p = 0.051; p = 0.081; p = 0.223; p = 0.005; p = 0.184, respectively).

Expression stabilities of the RNU48, let-7a and miR-16 were evaluated using NormFinder which is an accessible and widely used algorithm to determine the optimal normalization gene between a set of candidates. Same data from analysis in Fig. 1 was used and NormFinder determined that the best gene for normalization is let-7a (stability value 0.014). RNU48 and miR-16 got higher values (0.045 and 0.032, respectively) that correspond to lower stability. A combination of RNU48 and miR-16 as normalization genes got a better stability value of 0.014.

It should be noted that a good EC should also normalize the variability and reduce standard deviation (SD) that can be caused by many inconspicuous factors like patient age, gender or metabolic condition. It can be seen from Fig. 1 that some ECs that we have analyzed reduces miRNA expression variability better that others. For example miRNA expression normalized with miR-16 or let-7a has substantialy lower variability than miRNA expression normalized with RNU48. Therefore, researches should also notice and take into account the degree of variability when selecting the ECs for their study.

### Differential stability of target miRNAs and ECs

**Fig. 2:**
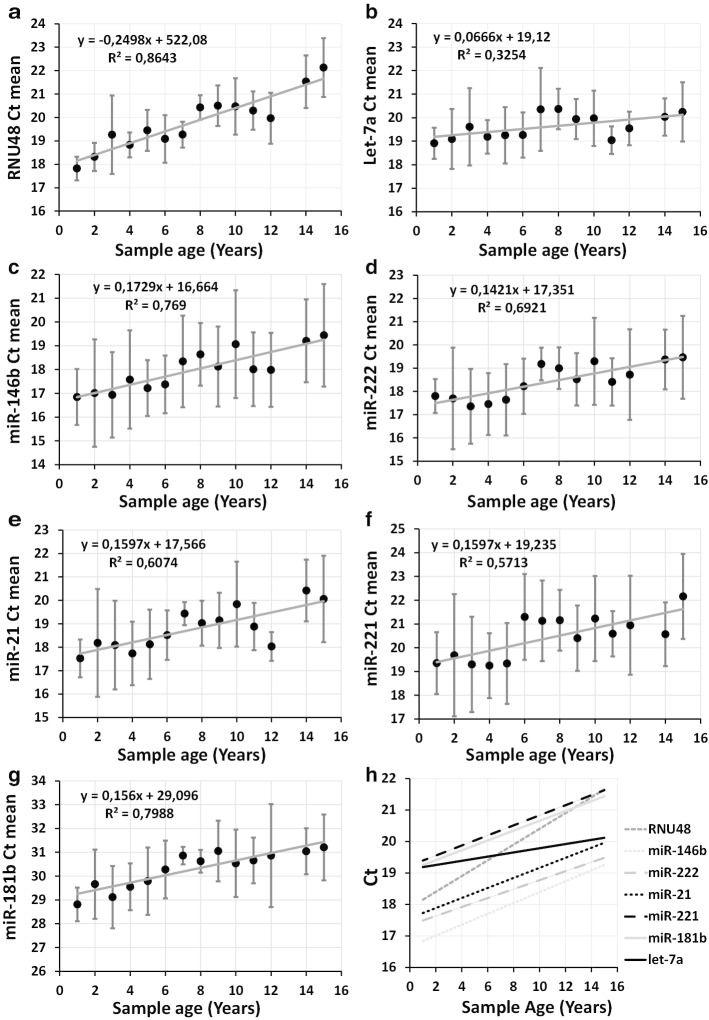
Differences in endogenous controls and target miRNAs degradation rate. **a**–**g** Correlation analysis between PTC FFPE tissue sample age and detected RNU48, let-7a, miR-146b, miR-222, miR-21, miR-221, miR-181b cycle of threshold (Ct). **h** Combined regression lines of miR-146b, miR-222, miR-21, miR-221, miR-181b and control RNU48, let-7a Ct values against sample age. For this graph miR-181b regression line is lowered by 10 Ct

To further analyze the association between PTC FFPE sample age and the levels of detected miR-146b, miR-222, miR-21, miR-221, miR-181b and control RNU48, let-7a expression we have used the linear regression models. More PTC FFPE samples were included in this experiment (n = 400) and their storage time varied from 1 to 15 years (samples were prepared between 2003 and 2017). We continued this experiment with two selected ECs—RNU48 and let-7a—because RNU48 is one of the most popular ECs in general and let-7a is one of the few miRNA-type controls used for miRNA research in PTC tissues. Also, we have demonstrated that let-7a seemed to normalize miRNA expression to sample age the best. Figure 2 shows how the detected Ct for individual miRNA or EC changed depending on the sample age: all analyzed miRNAs and ECs slowly but statistically significantly (p < 0.001) degraded over the years. However, it was not obvious whether the degradation rates really differ between the target miRNAs and the ECs.

To better evaluate the differences in target miRNAs, RNU48 and let-7a degradation rate we combined regression lines from Fig. 2a–g and also tested whether the linear regression slope coefficients are statistically significantly different between the regression lines (Fig. 2h and Table 1). Statistical analysis demonstrated that the slopes of target miRNA regression lines are very similar (p > 0.43), while the slopes of either RNU48 or let-7a regression lines differ substantially from the others (p < 0.08). This means that over the years the target miRNAs degrade at a very similar rate, but the degradation of ECs is different. As shown in Fig. 2h, the rate of RNU48 degradation is significantly more rapid than that of target miRNAs while let-7a degradation rate is slower. Therefore, when these ECs are used to normalize the detected miRNA expression profiles in PTC FFPE tissues stored for a different period of time, false results can be obtained.

**Table 1:** P-values from the test whether miR-146b, miR-222, miR-21, miR-221, miR-181b and control RNU48, let-7a regression line slope coefficients are same or significantly different

	let-7a	miR-146b	miR-222	miR-21	miR-221	miR-181b
RNU48	*0.00011*^a^	0.06368	*0.01189*	0.06704	0.07882	*0.01662*
let-7a		*0.01149*	0.06401	0.05469	0.0667	*0.01948*
miR-146b			0.43156	0.77972	0.7936	0.63587
miR-222				0.70195	0.72159	0.70195
miR-21					1	0.92118
miR-221						0.92118

^a^Italic numbers indicate statistically significantly different slopes of the regression lines

An important observation from this study is that when the data are normalized using the EC that degrades at a different rate then the target miRNAs the results of miRNA expression obtained with each separate EC are completely different. When we compared miRNA expression pattern from old FFPE blocks with miRNA expression in fresher specimens, we obtained statistically significant differences for the majority of miRNAs (Fig. 1). This indicates that RNU48 degrades faster than target miRNAs, which means that the older the sample the less RNU48 is detectable. Therefore, in older FFPE blocks higher miRNA expression levels are measured. In contrast, there is an opposite effect with EC genes (like let-7a) that are more stable than the miRNAs of interest. It means that if miRNA expression levels between groups are compared when the FFPE samples for these groups are of different age, there is a high risk to obtain inaccurate and unreliable results.

In general, probably every miRNA and the EC gene have its own individual degradation rate. These differences in degradation rate might be caused by differences in length (snoRNAs are usually more than double in length compared to miRNAs), their primary sequence, function, placement in the cell and association with proteins. Therefore, this differential storage-dependent transcript degradation should be taken into account when planning the experiments. It also means that there should be a more careful interpretation of miRNA profiling data when working with tissue samples of different age. Also, more research is needed to find reference genes that would not only be stable in the tissue of interest but would also behave like target miRNAs during a prolonged storage in FFPE tissue specimens. Moreover, as it might be quite impossible to find such perfect reference genes we would suggest combining several reference genes with best properties to improve RQ-PCR data accuracy.

## Conclusions

Commonly used endogenous controls (RNU48 and let-7a) have significantly different degradation rates than target miRNAs in FFPE papillary thyroid carcinoma tissues. This affects both detected miRNA levels and the obtained differences between study groups when working with samples of different age. Therefore, we expect that this study will be helpful for researchers searching for proper ECs that would assure the most accurate data for miRNA expression studies.

## Data Availability

This manuscript reports original data that was acquired in biomedical study of genetic and apigenetic biomarkers for PTC recurrence prediction. The datasets used and/or analysed during the current study are available from the corresponding author on reasonable request.

## Abbreviations

MiRNAs: MicroRNA
RQ-PCR: Real-time quantitative polymerase chain reaction
EC: Endogenous control
PTC: Papillary thyroid carcinoma
FFPE: Formalin-fixed, paraffin-embedded
RIN: RNA integrity number
RT: Reverse transcription
snoRNA: Small nucleolar RNA
SD: Standard deviation
